# Wideband and Large Angle Electromagnetically Induced Transparency by the Equivalent Transmission Line in a Metasurface

**DOI:** 10.1038/s41598-019-51967-3

**Published:** 2019-11-01

**Authors:** Renxia Ning, Dekai Li, Tianlong Yang, Zhenhai Chen, Hongwen Qian

**Affiliations:** 1 School of Information Engineering, Huangshan University, Huangshan, 245041 China; 2 Engineering Technology Research Center of Intelligent Microsystems, AnHui Province, 245041 China; 3 Fifty-eight China Electronic Science and Technology Corporation, Wuxi, 214035 China

**Keywords:** Optical physics, Optical physics

## Abstract

A classical structure for a U-shaped metasurface exhibiting a wideband and large angle electromagnetically induced transparency (EIT) effect in the terahertz range is proposed. One horizontal and two vertical strips, which represent the bright and dark modes, respectively, are created for the U-shaped structure. The finite integration time domain (FITD) and equivalent circuit method are compared with the EIT result. The EIT effect is affected by the length of the vertical bar and by the distance from the vertical bar to the symmetry axis. The results show that the asymmetry of the main structure in the x and y axes makes it easier to achieve the EIT effect. In addition, by changing the incident angle, the EIT effect always exists until the angle of the incidental electromagnetic wave is 85 degrees. These results have many potential applications for terahertz filtering, large-angle switching and sensors.

## Introduction

Electromagnetically induced transparency (EIT) is the quantum interference effect produced by the interaction between the coherent electromagnetic field and atomic level system^1^. When a coherent electromagnetic field acts on a pair of transition levels in dielectric atoms, the medium absorbs most of the incident field at its resonance frequency. This occurs if there is a strong coherence between one of the abovementioned transition energy levels and another atomic energy level. When the coupling field and the detection field satisfy certain conditions, the absorption of the medium in the detection field will appear as a depression at the position of the original absorption peak, that is, the atomic system detects the resonance frequency. The absorption of light decreases or even disappears. The phenomenon when the original opaque medium becomes transparent under the action of the electromagnetic field is called EIT.

However, the experimental conditions to realize the electromagnetically induced transparency are difficult to achieve and require ultra-low temperatures and high-intensity optical pumping^2^. Researchers have found that by designing special structures at room temperature, it is possible to imitate the EIT phenomenon in certain materials such as in waveguides^3–5^, electric circuits^6^ and metamaterials^7–9^. In recent years, metamaterials in the microwave^10^, terahertz (THz)^11^, infrared^12^ and optical bands^13^ that achieve the EIT effect attracted considerable attention. These materials are used in nonlinear optics^14^, slow light and optical storage^15,16^.

Currently, radiation mode coupling^17,18^, plasma hybridization^19,20^ and Fano resonance^21,22^ are used to produce the EIT effect. Recently, two coupled harmonic oscillators^23^ and the equivalent circuit method^24^ were used to explain the physical mechanism of the EIT. A metamaterial was designed that can support a full dark resonant state upon interaction with an electromagnetic beam. The results show that the EIT effect can be achieved in metamaterials in which the electromagnetic radiation interacts resonantly with mesoscopic oscillators rather than with atoms^25^. The advantage of the dual oscillator model is that it can analyze complex structures^26,27^; however, it cannot provide accurate EIT values^28^.

The circuit model of the metamaterial structure helps designers to understand the electromagnetic characteristics of the metamaterial based on the circuit parameters. In addition, the tuning of the metamaterial characteristics can be more comprehensively analyzed based on the circuit parameters. An equivalent circuit model for analyzing the metamaterial resonance is established. The results show that weak, critical and strong couplings exist in the model, which depend on the loss and coupling strength and exhibit different resonance characteristics^29^. The equivalent circuit analysis method is applicable to the models with simple structure and shape such as split-ring resonators (SRRs) and cut wire (CW). However, it is difficult to analyze complex models with equivalent circuit^30^. Moreover, in the abovementioned cases, it is difficult to calculate circuit parameters in the low terahertz range.

In the paper, one horizontal and two vertical strips, which represent the bright and dark modes, respectively, are created for a U-shaped structure. The classical structure for a U-shaped metasurface exhibiting a wideband and large angle electromagnetically induced transparency (EIT) effect in the terahertz range is proposed. We used the finite integration time domain (FITD) and equivalent circuit method to compared with the EIT result. The EIT effect is affected by the length of the vertical bar and by the distance from the vertical bar to the symmetry axis. The results show that the asymmetry of the main structure in the x and y axes makes it easier to achieve the EIT effect. Moreover, a large – incidental EIT effect is existed until the angle of the incidental electromagnetic wave is 85 degrees. These results have many potential applications for terahertz filtering, large-angle switching and sensors.

## Results and Discussion

**Figure 1:**
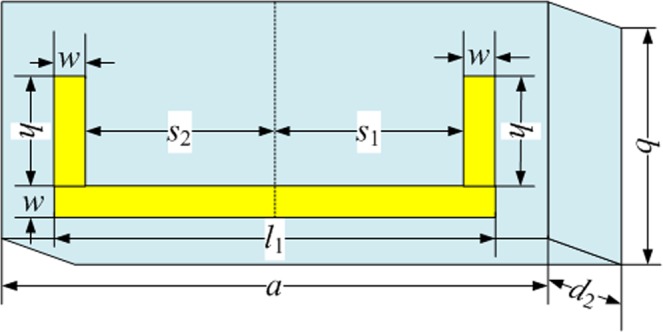
Schematic diagram and geometric parameters of the structure unit cell.

**Figure 2:**
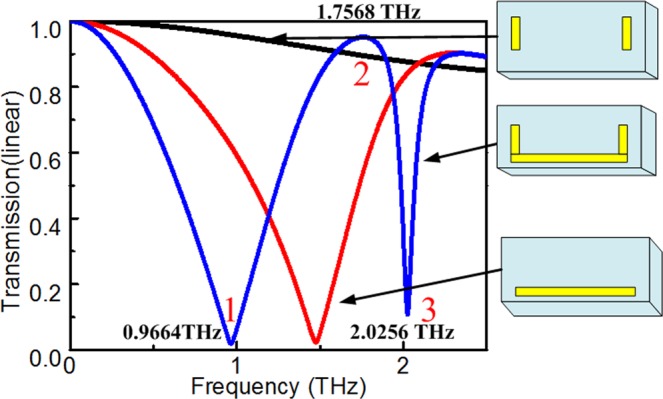
Transmission spectra for *a* = 100 μm, *b* = 38 μm, *d*_1_ = 0.02 μm, *d*_2_ = 12 μm, *l*_1_ = 80 μm, *w* = 4 μm, *l*_2_ = 20 μm, *w* = 4 μm, *h* = 26 μm, *s*_1_ = 38 μm, *s*_2_ = 38 μm.

To study the EIT of the metasurface structure, which consists of one horizontal and two vertical gold strips, we analyzed the transmission of the structure (shown in Fig. 1) with the following dimensions: *a* = 100 μm, *b* = 38 μm, *d*_1_ = 0.02 μm, *d*_2_ = 12 μm, *l*_1_ = 80 μm, *w* = 4 μm, *l*_2_ = 20 μm, *w* = 4 μm, *h* = 26 μm, *s*_1_ = 38 μm and *s*_2_ = 38 μm. The transmissions of only the horizontal strip, vertical strips, and the entire structure are shown as black, red, and blue lines in Fig. 2, respectively. The resonance of only the horizontal gold strip occurred at 1.5 THz as a bright mode (shown as a black line) and that of only the vertical strips did not resonate as dark modes (shown as a red line)^26^. Incident light can directly excite the dipole mode in the horizontal element, while the direct excitation of the dark mode in the vertical elements is not allowed. Clearly, the transmission spectrum of the metasurface structure shows an EIT window with a peak frequency of 1.7568 THz in addition to two dips positioned at 0.996 and 2.0256 THz (shown as a blue line in Fig. 2), which is similar to the EIT spectrum for atomic systems.

**Figure 3:**
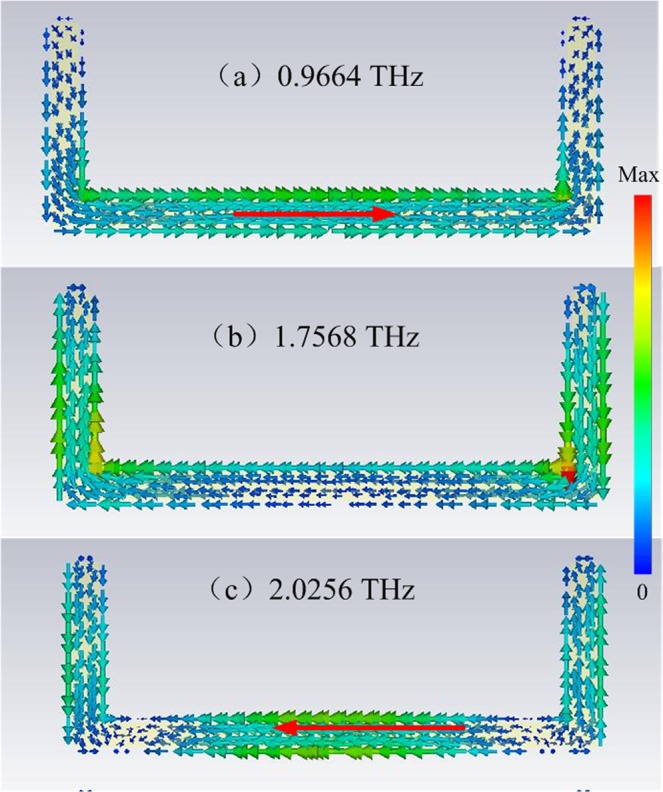
Distributions of current surface of the whole structure with coupled strips at (**a**) 0.996, (**b**) 1.7568, (**c**) and 2.0256 THz.

As shown in Fig. 2, the resonance dips of different structures are *ω*_whole1_ = 0.9664 THz, *ω*_whole2_ = 1.7568 THz, and *ω*_whole3_ = 2.0256 THz. Figure 3 shows the interference between the bright and dark modes and the distribution of the current surface at the frequencies of interest for the whole structure. Surface currents are directed in opposite directions at low (0.9664 THz) and high (2.0256 THz) frequencies. In addition, the surface current is weak at *ω*_whole2_ = 1.7568 THz. This implies that the incident light can directly excite the bright mode, and the dark mode can be indirectly activated via the coupling between the bright and dark modes. Then, the indirectly excited dark mode will couple back to the bright mode, which leads to a transparent window at 1.7568 THz.

**Figure 4:**
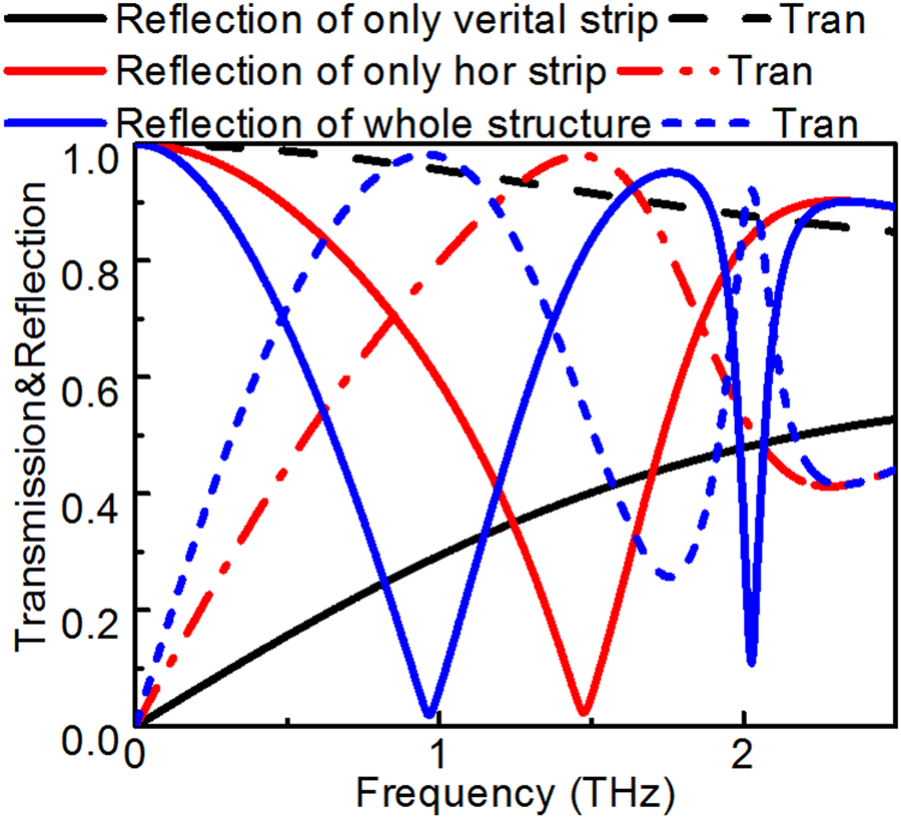
The variation with reflection and transmission spectra with different structure.

Of note, the vertical strips as dark modes lose energy at high frequencies (due to the decrease in transmission, shown in Fig. 4). As a dark mode, the transmission of the vertical bar decreases in the high-frequency band (existence of reflection), which results in the transmission of the valley increases in the high-frequency band (existence of reflection).

**Figure 5:**
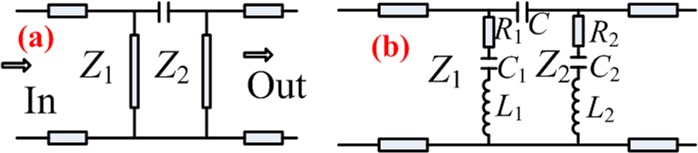
Transmission line equivalent circuit of the designed structure.

The equivalent circuit of the transmission lines (ECTL) model of EIT is shown in Fig. 5; the coupled harmonic oscillator is modeled by the 0.5*λ* transmission lines of *Z*_1_ and *Z*_2_. *Z*_1_ consists of *L*_1_ and *C*_1_, and *R*_1_ is used to simulate the bright harmonic oscillator; *Z*_2_ consists of *L*_2_ and *R*_2_, and *C*_2_ is used to simulate the dark harmonic oscillator. *C* represents the coupling between *Z*_1_ and *Z*_2_.

According to the transmission line theory, the input impedance of lossy transmission lines can be expressed as:

1 $${Z}_{in}={Z}_{0}\frac{{Z}_{L}+{Z}_{0}\,\tanh \,\gamma l}{{Z}_{0}+{Z}_{L}\,\tanh \,\gamma l},$$

where Z_0_ is the characteristic impedance of transmission lines, Z_L_ is the load impedance, *γ* is the attenuation factor, and l is the length of transmission lines.

In the designed structure, the load impedance, Z_L_, is considered infinitely large, and the impedance of the input terminal matches Z_0_ of 377 Ω. Thus, formula (1) can be approximated as:

2 $${Z}_{in}=\frac{{Z}_{0}}{\tanh \,\gamma l}={Z}_{0}\frac{1+j\,\tanh \,\alpha l\,\tan \,\beta l}{\tanh \,\alpha l+j\,\tan \,\beta l},$$

In formula (2),

3 $$\beta l=\pi +\frac{\pi \Delta \omega }{{\omega }_{0}},\,\tanh \,\alpha l\approx \alpha l,$$

Formula (4) is obtained from formulas (2) and (3).

4 $${Z}_{in}\approx \frac{{Z}_{0}}{\alpha l+j\pi \frac{\Delta \omega }{{\omega }_{0}}},$$

Compared with the input impedance of the resonant circuit,

5 $${Z}_{in}\approx \frac{R}{1+j\frac{2Q\Delta \omega }{{\omega }_{0}}},$$

Resonant transmission lines can be equivalent to resistors, inductors and capacitors in series or in parallel. Thus, the equivalent resistance, inductance and capacitance of a resonant transmission line are written as:

6 $$R=\frac{{Z}_{0}}{\alpha l},\,L=\frac{2{Z}_{0}}{\pi {\omega }_{0}},\,C=\frac{\pi }{2{\omega }_{0}{Z}_{0}},$$

**Figure 6:**
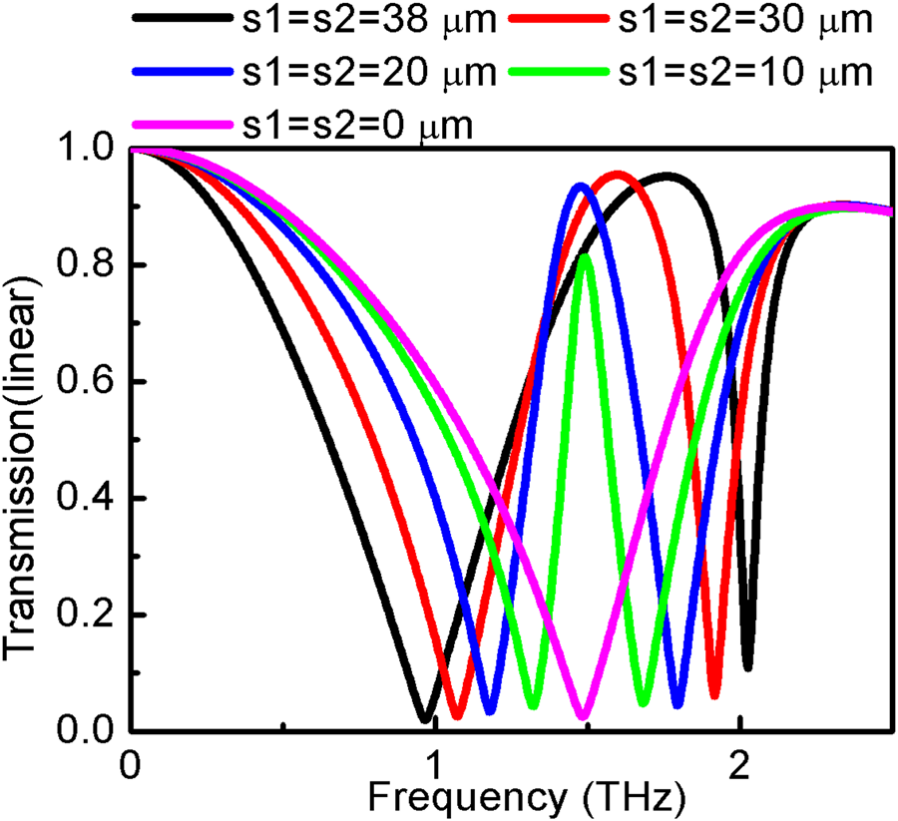
Compared with the transmission of different *s* at *a* = 100 μm, *b* = 40 μm, *d*_1_ = 0.02 μm, *d*_2_ = 12 μm, *l*_1_ = 80 μm, *h* = 26 μm, *w* = 4 μm, *l*_2_ = 20 μm, *s*_1_ = 0 μm, *s*_2_ = 38 μm.

Transmission spectra vary as a function of *s*, as shown in Fig. 6. Thus, the EIT effect can be achieved when *s* is not equal to zero. The transmission bandwidth increases with the increase in *s*. The EIT phenomenon disappears when s is zero due to two resonance frequencies that are close to each other. The resonance frequencies are extracted from the simulation results and are shown in Table 1. *Z*1, which consists of *L*1, *C*1 and *R*1, and *Z*2, which consists of *L*2, *R*2 and *C*2, are calculated according to formula (6) and are demonstrated in Table 1.

**Table 1:** Circuit parameter of equivalent transmission lines.

Parameters (μm)	*L*_1_ (pH)	*C*_1_ (fF)	*L*_2_ (pH)	*C*_2_ (fF)	*C* (fF)	*f*_1_ (THz)	*f*_2_ (THz)
*s*_1_ = *s*_2_ = 38	39.69772	0.688466	18.8768	0.327375	210	0.9632	2.0256
*s*_1_ = *s*_2_ = 30	35.56254	0.61675	19.98163	0.346535	290	1.0752	1.9136
*s*_1_ = *s*_2_ = 20	32.47015	0.56312	21.29949	0.369391	305	1.1776	1.7952
*s*_1_ = *s*_2_ = 10	28.93224	0.501763	24.15162	0.418854	315	1.3216	1.5832
*s*_1_ = *s*_2_ = 0	25.80781	0.447577	25.80781	0.447577	350	1.4816	1.4816

**Figure 7:**
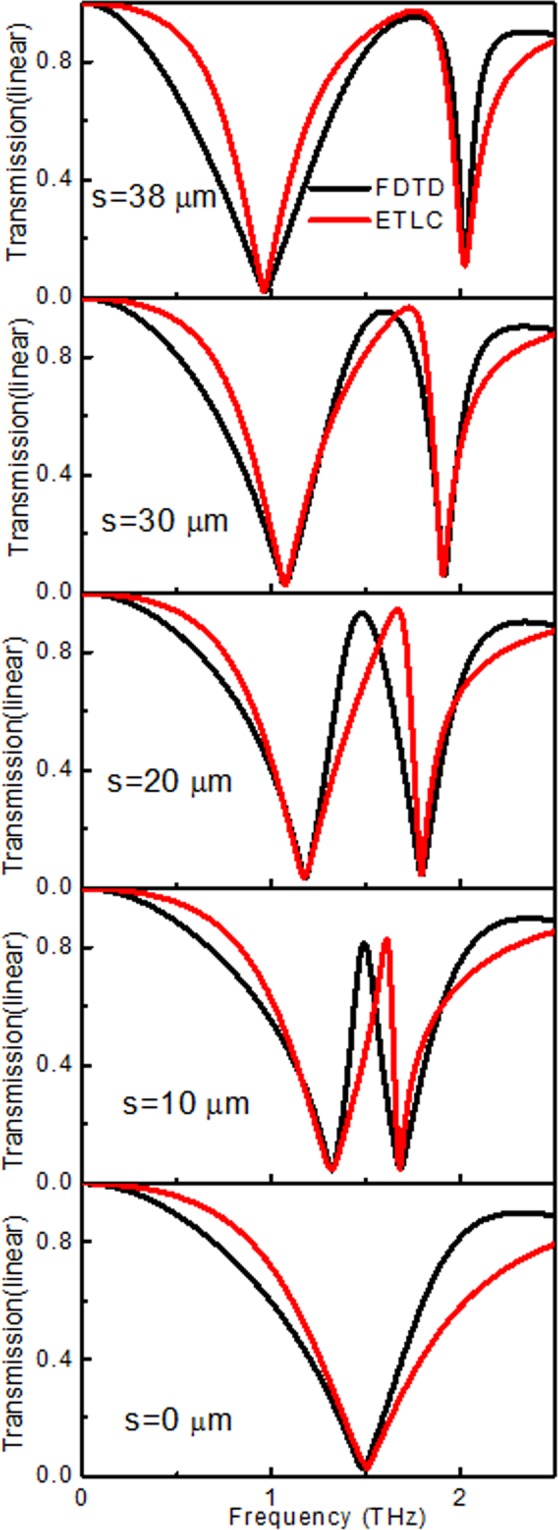
Comparison of transmission simulated by FITD methods (black line) and calculated by ECTL (red line) of different *s* varied from 0 to 38 μm.

Using the transmission line simulation method, the results for different *s* varied from FITD, as illustrated in Fig. 7. It is observed that the ECTL method and the obtained result are in good agreement with the FITD method in the low-frequency range. Of note, there is some difference between the equivalent transmission line of simulation results in the high-frequency range, especially for the quality factor Q. Parameter *b* can affect the EIT effect because the structure is asymmetric in the x and y axes, as shown in Fig. 7. The abovementioned parameter is an approximate equivalent, which leads to the discrepancies at high frequencies. Compared with the dual method results, the deviation from the equivalent transmission lines method does not considerably affect the EIT results.

**Figure 8:**
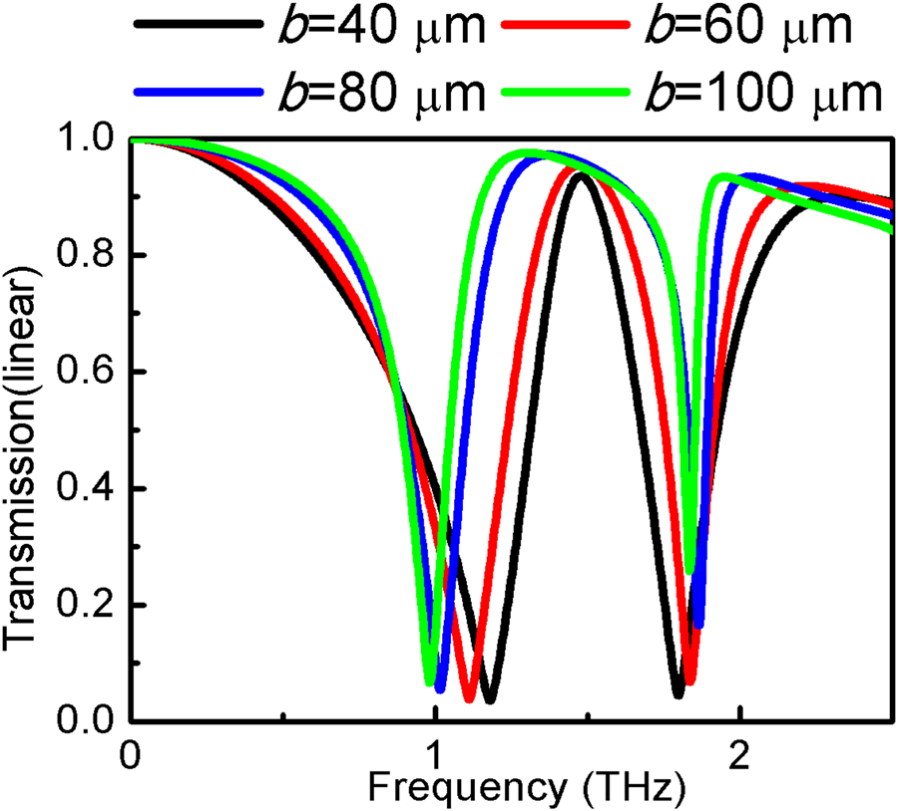
Transmission spectra of different *b* at *s*_1_ = *s*_2_ = 20 μm. *a* = 100 μm, *d* = 0.02 μm, *d*_2_ = 12 μm, *l*_1_ = 80 μm, *h* = 26 μm, *w* = 4 μm, *l*_2_ = 20 μm, *s*_1_ = *s*_2_ = 38 μm.

In Fig. 8, the transmission spectra in terms of frequency are depicted for various *b*. When *b* is large, the less clear the EIT is, especially at a high resonance frequency. In addition, redshift occurs at low frequency, and blueshift occurs at high frequency, which results in a wider EIT band. The EIT effect is least pronounced when x and y directions are equal in size, *a* = *b* = 100 μm. The results show that the worse the symmetry of the structure, the more pronounced are the EIT effects. This implies that the asymmetry of the main structure in the x and y axes makes it easier to achieve the EIT effect.

**Figure 9:**
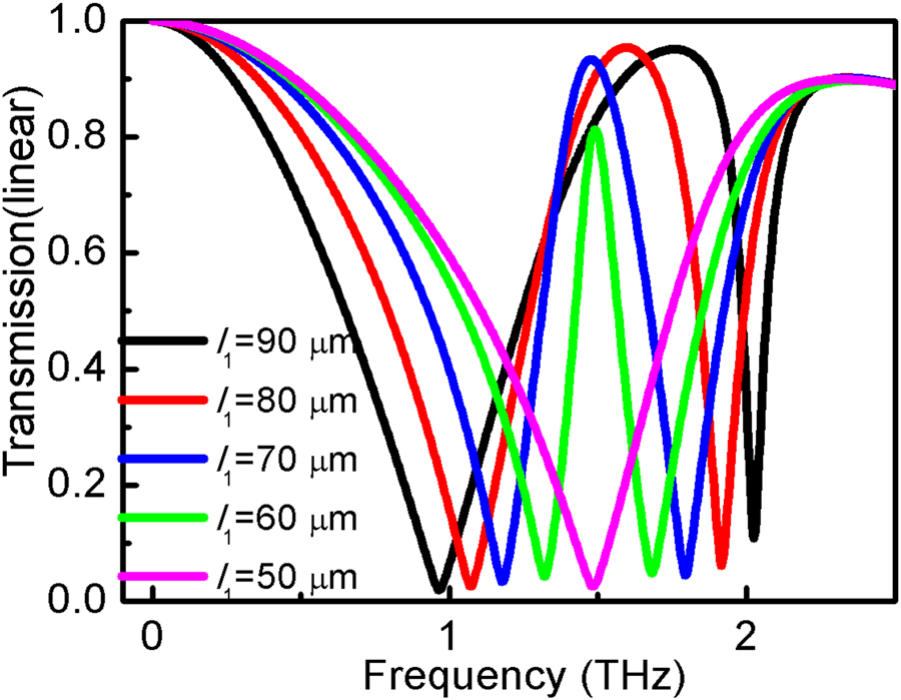
Variation of transmission of the structure at horizontal strip length *l*_1_.

Figure 9 shows the difference in transmission spectra as a function of the horizontal strip length. When the horizontal strip length, *l*_1_, increases, the transmission is more blueshifted at high frequency and less blueshifted at low frequency. It is clear that the horizontal strip can lead to resonance at high frequency. Thus, larger *l*_1_ can increase the wideband of the EIT effect.

**Figure 10:**
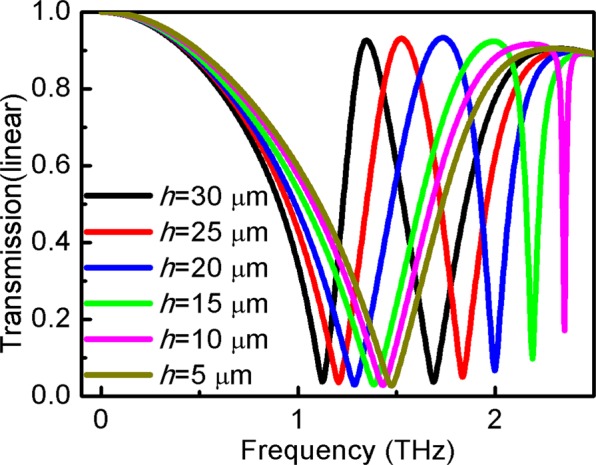
Variation of transmission of the structure at vertical strips length *h* at *s*_1_ = *s*_2_ = 20 μm.

Figure 10 shows the effect of the vertical strip length, *h*, on the EIT window. It is observed, that t, transmission at different frequencies, can vary in a wide range. Lower *h* can reduce the transmission band, which is clearly blue-shifted at high frequency. Figure 9 shows that the EIT phenomenon disappeared when *h* decreased by 5 μm at a certain frequency. This suggests that transverse strips as a bright mode cannot be coupled to the vertical strip as a dark mode due to its small size.

**Figure 11:**
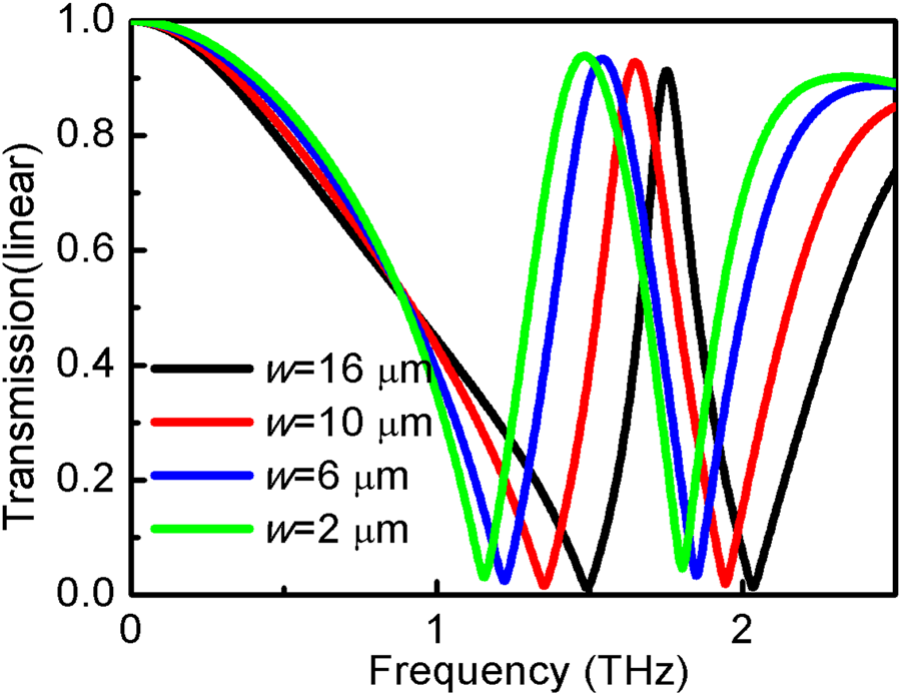
Variation of transmission of the structure at different strips width *w*.

Figure 11 shows the transmission coefficient as a function of *w* of the structure. It is observed that transmission behavior depends on the value of *w*. By increasing the value of *w*, the transmission window blueshifts.

**Figure 12:**
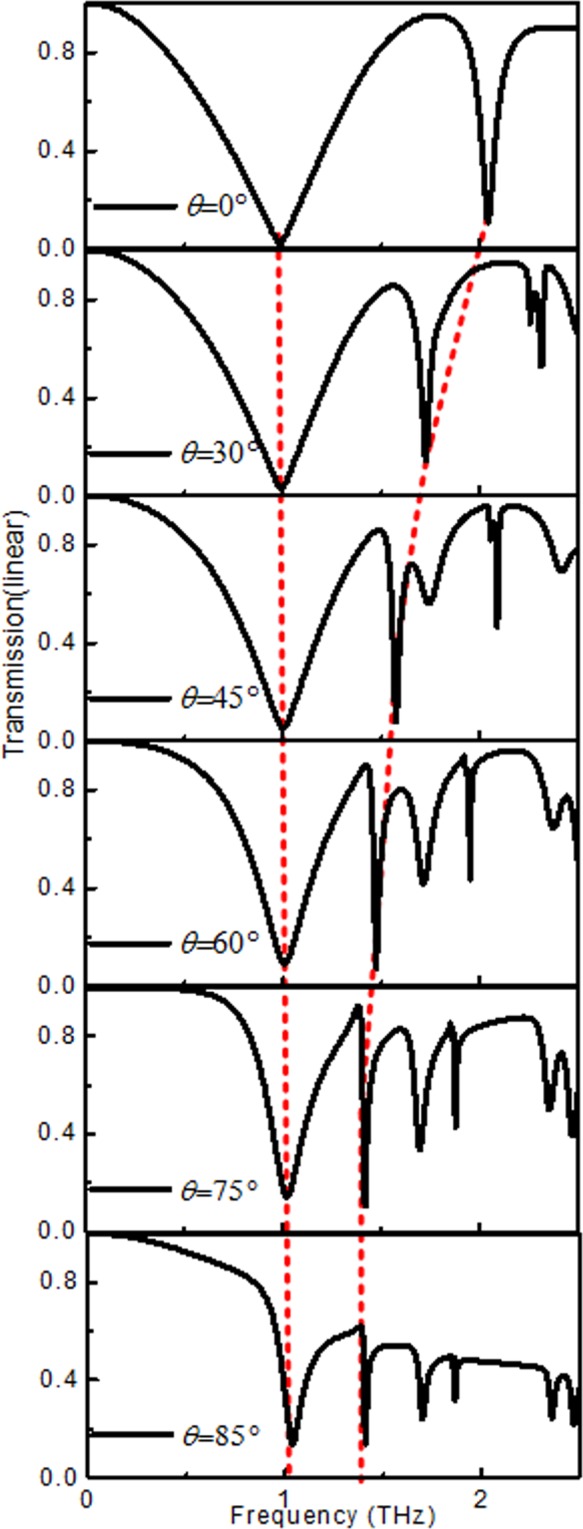
Transmission spectra of the multilayer structure for variation of *θ*.

Figure 12 shows the result for the oblique incident angle of the EM wave. The abovementioned figure clearly shows that larger incident angle results in band narrowing. The structure maintains the EIT effect in a wide range of incident angles, up to 85°. In addition, the transmission peak remains constant at low frequency and redshifts at high frequency. The abovementioned observation indicates that the large-angle EIT effect can be realized. This phenomenon can be explained in the following two ways. First, the smaller the loss is, the smaller the impact of the incident angle^31,32^. Second, the better the symmetry of the structure is, the smaller the influence of the incident angle^33,34^. This means that a larger THz filter and angle switch are applied using the designed structure.

## Conclusions

In this study, we investigated the classical structure for the U-shaped metasurface that exhibits a wideband and large incidence EIT effect via the FITD method in the THz range. The U-shaped metasurface structure consists of one horizontal and two vertical gold strips, which represent the bright and dark modes, respectively. To verify the numerical results, an equivalent transmission line was applied to compare it with the result obtained by the FITD method. The surface current distribution is used to explain the physical mechanism of the EIT, which is affected by the length of the vertical strip and by the distance from the vertical strip to the symmetry axis. In comparison with previous studies, more accurate model data were calculated using the described structure. In addition, by changing the incident angle, due to the absence of loss and symmetry in the x-direction, the EIT effect is always present up to 85 degrees. These results have many potential applications for terahertz filtering, slow light and large angle sensors.

## Methods

Using a unit cell of the metasurface structure, which consists of gold polyimide (shown in Fig. 1) and assuming that the electromagnetic (EM) wave propagates along the *z-*direction, we employ the FITD method for the normal incident angle (*θ* = 0°) of the electromagnetic wave with periodic boundary conditions. The *d*_1_ and *d*_2_ are the thickness of gold and polyimide, respectively. The designed structure is arranged along the x and y directions with a period of *a* × *b*. The gold layer on top of polyimide is composed of two vertical and one horizontal strip. The geometric parameters of the structure are as follows: *l*_1_ is the length of the horizontal strip, *w* is the width of the strips, *h* is the length of the vertical strips and *s*_1_ and *s*_2_ are the distances between the vertical strips and the center position, respectively. The dielectric permittivity of polyimide is 3.5, and the parameters for gold are shown in ref.^35^.
